# Are weekend inpatient rehabilitation services value for money? An economic evaluation alongside a randomized controlled trial with a 30 day follow up

**DOI:** 10.1186/1741-7015-12-89

**Published:** 2014-05-29

**Authors:** Natasha Kareem Brusco, Jennifer J Watts, Nora Shields, Nicholas F Taylor

**Affiliations:** 1 Physiotherapy Department, Faculty of Health Science, La Trobe University, Bundoora Campus, Bundoora, Victoria 3086, Australia; 2 Physiotherapy Services, Cabrini Health, 183 Wattletree Road, Malvern, Victoria 3144, Australia; 3 Deakin Health Economics, School of Health and Social Development, Faculty of Health, Deakin University, 221 Burwood Highway, Burwood, Victoria 3125, Australia; 4 Northern Health, Department of Allied Health, 1231 Plenty Rd, Bundoora, Victoria 3083, Australia; 5 Allied Health Clinical Research Office, Eastern Health, Level 2, 5 Arnold Street, Box Hill, Victoria 3128, Australia

**Keywords:** Rehabilitation, Economic evaluation, Randomized controlled trial, Allied health

## Abstract

**Background:**

Providing additional Saturday rehabilitation can improve functional independence and health related quality of life at discharge and it may reduce patient length of stay, yet the economic implications are not known. The aim of this study was to determine from a health service perspective if the provision of rehabilitation to inpatients on a Saturday in addition to Monday to Friday was cost effective compared to Monday to Friday rehabilitation alone.

**Methods:**

Cost utility and cost effectiveness analyses were undertaken alongside a multi-center, single-blind randomized controlled trial with a 30-day follow up after discharge. Participants were adults admitted for inpatient rehabilitation in two publicly funded metropolitan rehabilitation facilities. The control group received usual care rehabilitation services from Monday to Friday and the intervention group received usual care plus an additional rehabilitation service on Saturday. Incremental cost utility ratio was reported as cost per quality adjusted life year (QALY) gained and an incremental cost effectiveness ratio (ICER) was reported as cost for a minimal clinically important difference (MCID) in functional independence.

**Results:**

996 patients (mean age 74 (standard deviation 13) years) were randomly assigned to the intervention (n = 496) or the control group (n = 500). Mean difference in cost of AUD$1,673 (95% confidence interval (CI) -271 to 3,618) was a saving in favor of the intervention group. The incremental cost utility ratio found a saving of AUD$41,825 (95% CI -2,817 to 74,620) per QALY gained for the intervention group. The ICER found a saving of AUD$16,003 (95% CI -3,074 to 87,361) in achieving a MCID in functional independence for the intervention group. If the willingness to pay per QALY gained or for a MCID in functional independence was zero dollars the probability of the intervention being cost effective was 96% and 95%, respectively. A sensitivity analysis removing Saturday penalty rates did not significantly alter the outcome.

**Conclusions:**

From a health service perspective, the provision of rehabilitation to inpatients on a Saturday in addition to Monday to Friday, compared to Monday to Friday rehabilitation alone, is likely to be cost saving per QALY gained and for a MCID in functional independence.

**Trial registration:**

Australian and New Zealand Clinical Trials Registry November 2009 ACTRN12609000973213

## Background

There is debate about the potential advantages to the health system and broader community of providing health services on the weekend, such as those provided in intensive care and emergency departments [1,2]. The proposed advantages of providing weekend health services include improved clinical outcomes for patients [1] and fewer adverse events among those admitted on the weekend [3]. Resistance to providing weekend health services exists because of a lack of robust evidence that weekend services are safe and effective, opposition among health care professionals to embrace working on the weekend, and concerns that the health system needs to ensure weekday services (such as outpatient clinics and surgical lists) operate at full capacity before these services are expanded to the weekend [2].

Rehabilitation is an important part of the health system and involves specialized, coordinated, multidisciplinary care to restore functional independence in patients [4]. There is evidence that more intensive rehabilitation improves patient quality of life, functional independence and reduces length of stay [5]. Similarly, there is evidence that providing additional rehabilitation services on weekends improves patient quality of life, functional independence and is likely to reduce length of stay [6]. This may be related to increased physical activity among patients who received weekend rehabilitation services, reduced functional decline over the weekend because of increased physical activity, or changes in patient perceptions of no longer associating weekends with rest [6]. However, in spite of the available evidence, many rehabilitation facilities do not provide weekend services [7-9].

Before weekend rehabilitation services can be implemented routinely into practice it is important to find out whether they are cost effective from the perspective of the health service. Studies are needed to investigate if the increased cost of a weekend rehabilitation service is offset by savings within the inpatient episode of care. Economic evaluations of additional weekday inpatient rehabilitation programs have shown a lower cost per patient admission [10-12], but it is not known if providing weekend rehabilitation services is cost effective, especially since factors such as weekend penalty rates for clinical staff may make provision of such a service more expensive. In the debate on whether to provide weekend health services, the issue of who will pay and whether providing such services is cost effective is central [1,2].

The aim of this study was to undertake cost utility and cost effectiveness analyses alongside a multi-center, single-blinded randomized controlled trial with concealed allocation. The primary research question was to determine from a health service perspective if the provision of a rehabilitation service to inpatients on a Saturday in addition to Monday to Friday compared to Monday to Friday rehabilitation alone, was cost effective per quality adjusted life year (QALY) gained and for a minimal clinically important difference (MCID) in functional independence.

## Methods

### Research design

An economic evaluation was completed alongside a single-blinded randomized controlled trial that examined both the clinical effects and costs of providing a rehabilitation service (physiotherapy and occupational therapy) to inpatients on a Saturday in addition to Monday to Friday compared to Monday to Friday rehabilitation alone, for a mixed adult cohort of patients admitted for inpatient rehabilitation. The evaluation was completed from a health service perspective. The trial was registered with the Australian and New Zealand Clinical Trials Registry (ACTRN12609000973213) prior to patient recruitment. Full details of the protocol [13] as well as the clinical outcomes of the trial [6] have been published elsewhere. This economic evaluation report has been prepared with reference to the Consolidated Health Economic Evaluation Reporting Standards (CHEERS) Checklist (Additional file 1, [14]). The trial obtained ethics approval from the Eastern Health Research and Ethics Committee (E58 09/10) and La Trobe University Human Research Ethics Committee (FHEC10/14). All participants gave informed written consent.

### Intervention

Participants randomized to the intervention group received a rehabilitation service on a Saturday in addition to usual care Monday to Friday rehabilitation compared to the control group who received usual care Monday to Friday rehabilitation alone. Usual care typically consisted of interventions focused on task-specific training and discharge planning for independent living in the community. Patients were scheduled to receive about one hour of physiotherapy and one hour of occupational therapy each weekday as well as full nursing, medical and other allied health services. Over the weekend, usual care included full nursing care and a limited medical service to address high priority medical needs. In addition to usual care, the intervention group received rehabilitation therapy on a Saturday. They were scheduled to receive an hour each of physiotherapy and occupational therapy. Additional physiotherapy and occupational therapy were provided because they are the most frequent forms of therapy intervention during inpatient rehabilitation [15-17], with most inpatients at the included rehabilitation centers receiving daily physiotherapy and occupational therapy services on weekdays. To ensure continuity, the content of therapy provided at the weekend was decided by the participant’s usual weekday therapist and provided by a written handover.

### Participants and recruitment

From July 2010 until June 2011, eligible participants who were 18-years-old or older and admitted to one of two rehabilitation facilities were invited to participate. Patients with any orthopedic, neurological or other disabling condition were included. Patients were excluded if they did not give informed consent, were admitted for slow stream geriatric rehabilitation or if they were participating in another intervention trial.

Patients were randomized to the intervention or the control group using a concealed method, with 1:1 allocation. The block allocation sequence was generated electronically and assignments concealed in sequentially numbered, sealed, opaque envelopes. Only after the participant was enrolled in the trial and had completed baseline testing was group assignment made by opening the next envelope in the sequence.

### Sample size

Based on one of the primary outcome measures (length of stay) from a pilot study [18], a sample size of 712 participants was estimated [13]. To recruit this number of participants a recruitment period of 18 months was anticipated.

### Setting

The trial took place at two publicly funded metropolitan inpatient rehabilitation facilities with a combined total of 90 rehabilitation beds (providing multidisciplinary inpatient rehabilitation services in Melbourne, Australia). Prior to being accepted for inpatient rehabilitation, patients are typically assessed in an acute hospital as being able to participate actively in rehabilitation with the expectation that they will improve sufficiently to return to independent living in the community.

### Health service and therapy utilization

Patient length of stay for the rehabilitation admission was measured as the number of overnight stays in the rehabilitation unit, from the day of admission until the day of discharge, inclusive of any transfers to an acute ward to manage medical conditions. Any unplanned readmissions to the health service in the 30 day period immediately post discharge from rehabilitation were also recorded and included in the total length of stay. This period was considered relevant in the economic evaluation as readmissions account for a significant portion of all health care expenses and one third of all hospital readmissions are within one month of discharge [19].

Time units for utilization of allied health services during the rehabilitation admission were recorded on the hospital database. This captured Monday to Friday and Saturday interventions for physiotherapy and occupational therapy. Data source, unit definition and unit cost for each of these resources included in the economic evaluation are specified in Table 1.

**Table 1:** Costs included in the economic evaluation in 2010/2011 $AUD

	**Data source**	**Unit**	**Unit cost**
Itemized rehabilitation costs (excluding acute inpatient care)			
PT in rehabilitation			
PT Monday to Friday service	EBA [20] plus 25% loading	Per minute	$0.68
PT Saturday service	EBA [20] plus 50% loading	Per minute	$0.82
PT non salaries and wages (equipment)	PT 2010 health service budget^a^	Per day	$1.79
PT capital costs (space)	Health service	Per day	$3.51
OT in rehabilitation			
OT Monday to Friday service	EBA [20] plus 25% loading	Per minute	$0.68
OT Saturday service	EBA [20] plus 50% loading	Per minute	$0.82
OT non salaries and wages (equipment)	OT 2010 health service budget^α^	Per day	$1.79
OT capital costs (space)	Health service	Per day	$3.51
Other allied health cost	Health service	Per day	$69.39
Nursing salaries and wages	Health service	Per day	$410.13
Other costs^b^	Health service	Per admission	Variable
Health service capital cost (space)	Health service	Per day	$7.03
Acute ward costs during rehabilitation, total	Health service	Per admission	Variable
Readmissions 30 days post discharge cost, total	Health service	Per admission	Variable

^a^obtained from Eastern Health Physiotherapy and Occupational Therapy budgets for the 2010/2011 financial year; ^b^coronary care, intensive care and emergency departments, imaging, surgical, non-surgical, pathology, pharmacy, prosthetics, theatre and other health service costs, EBA*,* enterprise bargaining agreement; OT, occupational therapy; PT, physiotherapy.

### Cost of inpatient rehabilitation

Cost data were collected from the health service and covered two financial years; 2010/2011 and 2011/2012. Data from the 2011/2012 financial year (6% of sample) were discounted at a rate of 3.5%, consistent with the corresponding national Consumer Price Index [21], so all data are reported in 2010/2011 Australian dollars (AUD$).

Total cost included the cost of the rehabilitation admission, acute care costs during the rehabilitation admission, and the cost of any readmission during the 30 day period post discharge from rehabilitation (Table 1). Cost data were obtained from the inpatient clinical costing system from each site. However, as an average cost is used to attribute costs to all components of care (which would not take account of the higher costs of Saturday allied health services), the allied health and nursing components were modelled for each participant based on the actual utilization of these services. For physiotherapy and occupational therapy, the time unit was a minute and the unit cost the rate per minute for a therapist (mid-range seniority, inclusive of 25% on-costs). For all other allied health and nursing costs, the total length of stay was multiplied by a per diem rate. The costs for weekend therapy services were increased by 25% to allow for penalty loading. These costs were substituted into the clinical costing data so that a modelled totalled cost could be determined for each participant. Capital costs were included based on the length of stay for the ward component and on a daily rate for each day of use for the physiotherapy and occupational therapy departmental space.

### Outcomes measures for inpatient rehabilitation

The EuroQol (EQ-5D-3L) questionnaire [22] was converted to a health related quality of life utility score using UK utility weights based on the time trade-off (TTO) method [23,24]. Functional independence was measured using the FIM [25] administered by credentialed assessors. FIM scores can range from 18 (lowest function) to 126 (highest function). An increase in the FIM score of 22 points is considered to be a MCID in functional independence [26]. Both outcome measures were administered on admission (baseline) and on discharge from rehabilitation by assessors blind to group allocation.

### Statistical analysis

Outcome and cost data were analyzed according to the intention-to-treat (ITT) principle with multiple imputation used to account for missing outcome data [27-29] but was not required for cost data. Between group differences in the quality of life utility score and the functional independence score were calculated with analysis of covariance (ANCOVA) of the discharge score using the baseline score as covariate [28,29]. Mean cost difference was determined between the two groups using an independent *t*-test to report statistical significance [30]. Incremental cost effectiveness ratios (ICERs) were determined for both the quality of life utility score and the functional independence score using adjusted mean differences between groups from admission to discharge, derived from ANCOVA for outcomes and the mean difference between groups for cost.

Confidence intervals around the individual ICERs for the quality of life utility score and the functional independence score were calculated using the bootstrap method (5,000 repetitions), the change in outcome measures from admission to discharge and total cost [31]. Individual ICERs were used to generate the confidence ellipses and the cost effectiveness acceptability curves (CEACs), using the central limit theorem [31]. CEACs illustrate the probability that the intervention was cost effective compared to the control group, across a range of willingness to pay values per QALY gained and for the MCID in functional independence. While the ellipses provide important information relating to the statistical significance of the individual intervention under review, the CEACs provide a broader universal measure that allows comparison between interventions.

The ICER for the quality of life utility score represents the cost per QALY gained. The ICER for a one point change in the functional independence score was multiplied to report the cost difference for a 22 point change in the functional independence score, representing the cost difference for a MCID in functional independence. The likelihood of achieving a 22 point MCID in functional independence was reported as a relative risk (RR) between groups. The rate of change per day was calculated for quality of life utility and functional independence scores by taking the difference between admission and discharge for each outcome and dividing by each patient’s length of stay. Patient readmissions 30 days post discharge from rehabilitation were reported as a relative risk (RR) between groups.

Analyses were completed using IBM SPSS Statistics Version 21 [32] and customized software in Microsoft Excel [31]. All statistical tests were conducted at 5% level of significance and 95% confidence intervals (CI) unless otherwise stated.

### Sensitivity analysis

A sensitivity analysis was performed by removing the penalty loading for Saturday physiotherapy and occupational therapy wage rates. The reason for this is because the working week and the weekend may differ by country, for example in some settings and cultures Saturday is regarded as a normal working day [33]. Consideration was given to including a sensitivity analysis based on inflation rates. However, this was not completed as 94% of the cost data fell within 2010/2011 financial year.

## Results

A total of 996 patients were randomized to the control group (n = 500) or the intervention group (n = 496) with the flow of the patients through the trial reported elsewhere [6]. Recruitment rates were higher than originally expected and the project steering committee decided to stop recruitment earlier than planned as it appeared that the target sample size would be reached prior to 18 months. Without any interim analyses being performed, it was decided to stop recruitment at 12 months.

### Participants

Patients had a mean age of 74 years (standard deviation (SD) 13) and 631 (63%) were women. The groups appeared similar for diagnosis and co-morbidities (Table 2). There were some missing data for the EQ-5D-3L questionnaire at baseline (n = 54, 5%) and at discharge (n = 94, 9%). The main reason for missing EQ-5D-3L data was reduced patient cognition. There was also a small amount of missing data for functional independence score at baseline (n = 1, <1%) and discharge (n = 4, <1%).

**Table 2:** Baseline characteristics

**Characteristic**	**Randomized (n = 996)**	
	**Intervention (n = 496)**	**Control (n = 500)**
Age (years), mean (SD)	75 (13)	74 (13)
Gender, number males (%)	189 (38)	176 (35)
Diagnosis category, number (%)		
Stroke	81 (16)	79 (16)
Other neurological conditions	19 (4)	24 (5)
Orthopedic conditions	284 (57)	297 (59)
Pain syndromes	24 (5)	19 (4)
Cardiac/Pulmonary	25 (5)	23 (5)
Other disabling impairments	63 (13)	58 (12)
Charlson co-morbidity index [34], mean (SD)	1 (1)	1 (1)

SD, standard deviation.

### Health service and therapy utilization

Participants in the intervention group received on average an additional 53 minutes of rehabilitation therapy (95% CI 31.0 to 74.1) per week compared to the control group (Table 3).

**Table 3:** Mean (SD) of groups and mean (95% CI) difference between groups for health service utilization

	**Groups**		**Difference between groups 95% CI**
	**Intervention (n = 496)**	**Control (n = 500)**	**Intervention minus control**
Initial rehabilitation admission (days)	21.2 (15.7)	23.1 (20.2)	-1.9 (-4.1 to 0.4)
Rehabilitation ward (days)	20.9 (15.3)	22.7 (19.9)	-1.8 (-4.0 to 0.4)
Acute ward during rehabilitation (days)	.2 (1.8)	.3 (2.2)	-0.1 (-0.3 to 0.2)
30 days post discharge readmission(s) (days)	4.6 (16.2)	5.6 (18.9)	-1.0 (-3.2 to 1.2)
Total length of stay for initial admission and 30 day post discharge readmission(s) (days)	25.8 (25.1)	28.7 (32.2)	-2.9 (-6.5 to 0.7)
Initial rehabilitation admission therapy utilization			
PT in rehabilitation			
Monday to Friday (minutes)	826.4 (761.1)	863.29 (836.4)	-36.9 (-136.4 to 62.5)
Saturday (minutes)	118.5 (116.2)	0.7 (5.3)	117.8 (107.6 to 128.1)*
Total (minutes)	944.9 (858.0)	864.0 (836.7)	80.9 (-24.5 to 186.2)
OT in rehabilitation			
Monday to Friday (minutes)	506.7 (613.8)	533.0 (594.2)	-26.3 (-101.4 to 48.8)
Saturday (minutes)	94.8 (101.1)	0.3 (3.7)	94.5 (85.7 to 103.5)*
Total (minutes)	601.5 (690.9)	533.3 (594.8)	68.3 (-11.9 to 148.5)
Average therapy per week (minutes)	502.6 (167.2)	450.0 (179.3)	52.6 (31.0 to 74.1)*

*statistically significant at a *P* ≤0.05 level; OT, occupational therapy; PT, physiotherapy.

Mean hospital length of stay during the initial rehabilitation admission and subsequent admissions in the 30 day period post discharge from rehabilitation was 25.8 days (SD 25.1) for the intervention group and 28.7 days (SD 32.2) for the control group, with a mean difference of -2.9 days (95% CI -6.5 to 0.7) in favor of the intervention group (Table 3).

The initial rehabilitation admission was 21.2 days (SD 15.7) for the intervention group and 23.1 days (SD 20.2) for the control group, with a mean difference of -1.9 days (95% CI -4.1 to 0.4) in favor of the intervention group. In the 30 day period post discharge from rehabilitation, readmission average length of stay was 4.6 days (SD 16.2) in the intervention group compared to 5.6 days (SD 18.9) in the control group, with a mean difference of -1.0 day (95% CI -3.2 to 1.2) in favor of the intervention group (Table 3). There was no difference in the 30 day readmission rate between groups (RR = 1.01, 95% CI 0.95 to 1.07), with 19% (n = 92) of the patients in the intervention group and 19% (n = 95) of the patients in the control group readmitted during this period.

### Cost of inpatient rehabilitation

The mean total cost of the rehabilitation episode (including 30 day readmission costs) was $15,859 (SD 13,992) for the intervention group and $17,532 (SD 17,108) for the control group, with a mean cost difference of -$1,673 (95% CI -3,618 to 271) in favor of the intervention group (Table 4).

**Table 4:** Mean (SD) of groups and mean (95% CI) difference between groups for health service costs

	**Groups**		**Difference between groups 95% CI**
	**Intervention (n = 496)**	**Control (n = 500)**	**Intervention minus control**
Initial rehabilitation admission cost	13,320 (9,894)	14,275 (11,945)	-955 (-2,320 to 409)
Rehabilitation costs (see below), total	13,049 (9,506)	13,951 (11,597)	-902 (-2,221 to 417)
Acute ward costs during rehabilitation, total	271 (1,853)	324 (1,952)	-53 (-290 to 183)
Readmissions 30 days post discharge cost, total	2,539 (8,252)	3,257 (9,711)	-718 (-1,839 to 403)
Total cost for initial admission and 30 day post discharge readmission(s)	15,859 (13,992)	17,532 (17,108)	-1,673 (-3,618 to 271)
Itemized rehabilitation costs (excluding acute)	13,049 (9,506)	13,951 (11,597)	-902 (-2,221 to 417)
PT in rehabilitation	759 (662)	685 (637)	74 (-6 to 155)
PT Monday to Friday service	563 (518)	587 (564)	-24 (-91 to 43)
PT Saturday service	97 (95)	1 (4)	96 (88 to 105)*
PT non salaries and wages (equipment)	37 (27)	41 (35)	-3 (-7 to 1)
PT capital costs (space)	63 (46)	57 (49)	6 (0 to 12)
OT in rehabilitation	526 (542)	463 (468)	62 (-1 to 125)
OT Monday to Friday service	345 (418)	362 (400)	-17 (-68 to 34)
OT Saturday service	77 (83)	0 (3)	77 (70 to 85)*
OT non salaries and wages (equipment)	41 (30)	44 (38)	-3 (-8 to 1)
OT capital costs (space)	63 (46)	57 (49)	6 (0 to 12)
Other allied health cost	1,450 (1,061)	1,572 (1,365)	-122 (-274 to 30)
Nursing salaries and wages	8,568 (6,270)	9,289 (8,068)	-721 (-1,620 to 178)
Other costs^a^	1,600 (1,452)	1,783 (1,747)	-183 (-383 to 17)
Health service capital cost (space)	147 (107)	159 (138)	-12 (-28 to 3)

^a^coronary care, intensive care and emergency departments, imaging, surgical, non-surgical, pathology, pharmacy, prosthetics, theatre and other health service costs; *statistically significant at a *P* ≤0.05 level. OT, occupational therapy; PT, physiotherapy.

The initial rehabilitation admission cost was on average $13,320 (SD 9,894) for the intervention group and $14,275 (SD 11,945) for the control group, with a mean cost difference of -$955 (95% CI -2,320 to 409) in favor of the intervention group. In the 30 day period post discharge from rehabilitation, the average cost of admissions back to the health service was $2,539 (SD 8,252) for the intervention group and $3,257 (SD 9,711) for the control group, with a mean cost difference of -$718 (95% CI -1,839 to 403) in favor of the intervention group (Table 4).

### Outcomes measures for inpatient rehabilitation

Participants in the intervention group had a significantly higher change in their health related quality of life utility index score between admission and discharge compared to the control group (mean difference 0.04, 95% CI 0.01 to 0.07) (Table 5) and the mean rate of change per day was also significantly higher compared to the control group (mean difference 0.004, 95% CI 0.001 to 0.008). Participants in the intervention group also had a significantly higher change in functional independence score between admission and discharge (mean difference 2.3, 95% CI 0.5 to 4.1) compared to the control group (Table 5) and the mean rate of change per day was also significantly higher (mean difference 0.16, 95% CI 0.04 to 0.28) compared to the control group.

**Table 5:** Mean (SD) of groups and mean (95% CI) difference between groups for functional status and quality of life

**Outcome**	**Groups**				**Difference between groups 95% CI**
	**Admission**		**Discharge**		
	**Intervention (n = 496)**	**Control (n = 500)**	**Intervention (n = 496)**	**Control (n = 500)**	**Intervention minus control**
EQ-5D-3L (utility weight)	0.32 (0.35)	0.37 (0.35)	0.65 (0.28)	0.62 (0.28)	0.04 (0.01 to 0.07)*
Total FIM score	83.8 (19.2)	83.8 (19.9)	105.9 (18.4)	103.5 (20.1)	2.3 (0.5 to 4.1)*

*indicates *P* <0.01. EQ-5D-3L, EuroQOL questionnaire. FIM, functional independence measure.

### Incremental cost effectiveness ratios

The incremental cost utility ratio showed a cost saving of $41,825 (95% CI -2,817 to 74,620) per QALY gained for the intervention group compared to the control group. The incremental cost effectiveness ratio showed a cost saving of $727 (95% CI -159 to 3,845) for a one point change in the functional independence score for the intervention group compared to the control group. This equates to a cost saving of $16,003 (95% CI -3,074 to 87,361) for a MCID in functional independence for the intervention group compared to the control group. To put these outcomes in context, patients in the intervention group were 17% more likely to achieve a MCID in functional independence (RR 1.17, 95%CI 1.03 to 1.34) at discharge compared to those in the control group.

**Figure 1:**
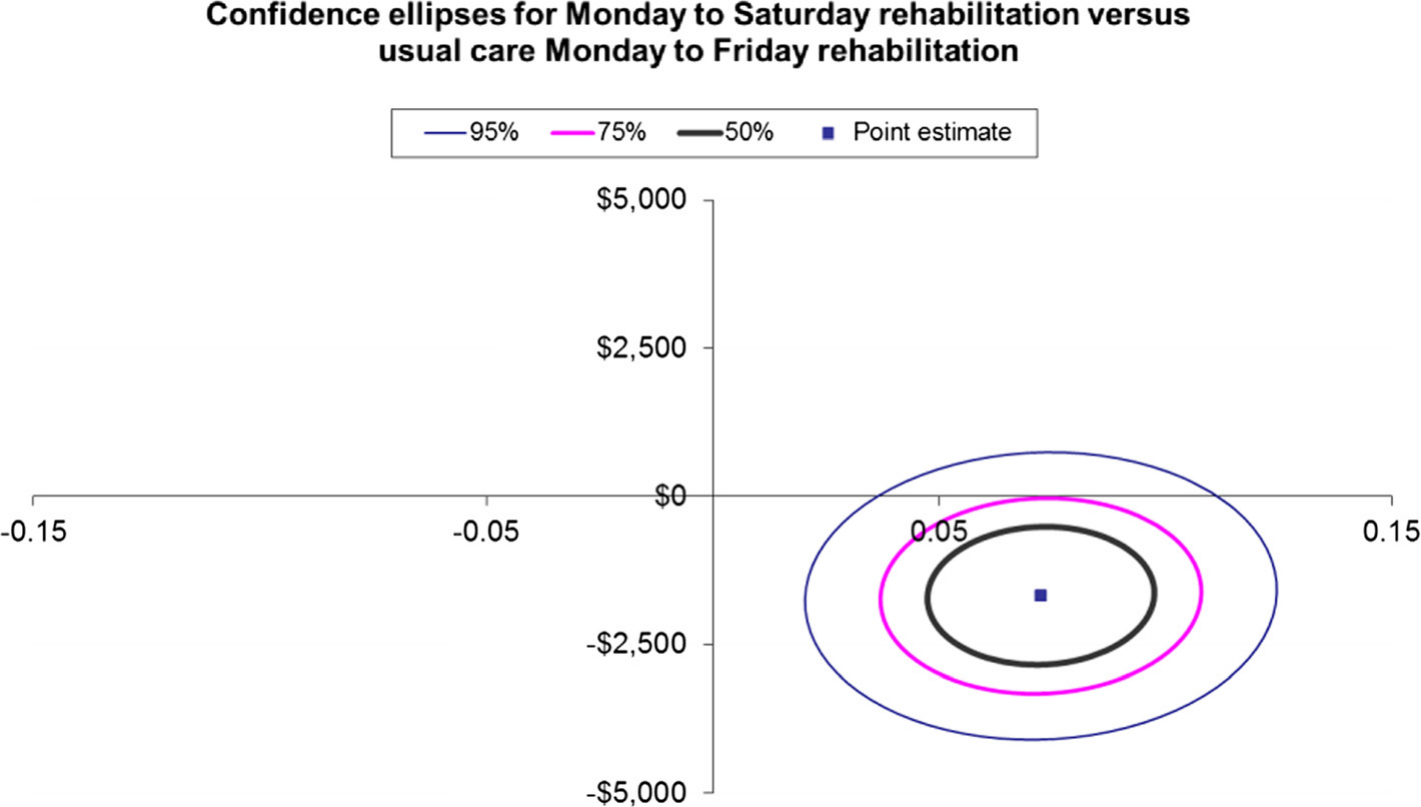
Confidence ellipses for Monday to Saturday rehabilitation versus usual care Monday to Friday rehabilitation for the incremental cost (vertical axis AUD$2010/11) per quality adjusted life year (QALY) gained (horizontal axis).

**Figure 2:**
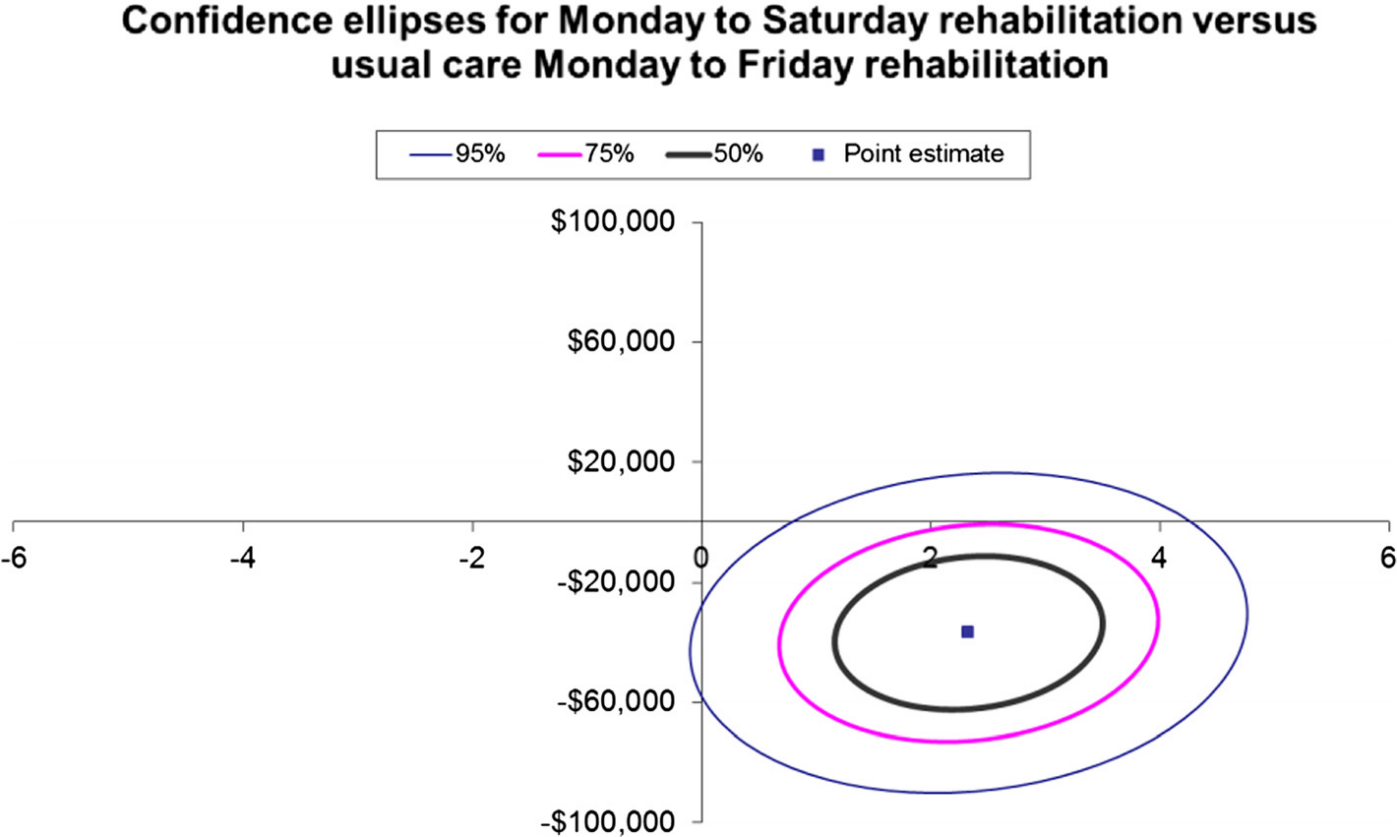
Confidence ellipses for Monday to Saturday rehabilitation versus usual care Monday to Friday rehabilitation for the incremental cost (vertical axis AUD$2010/11) per minimal clinically important difference (MCID) in function gained (horizontal axis).

The ICER ellipses for confidence intervals (50%, 75% and 95%) are presented in Figures 1 and 2 for a QALY gained and for a MCID gained in functional independence, respectively. The confidence ellipses show that all the 50% and 75% confidence intervals sit within the bottom right quadrant of the cost effectiveness plane and only a small portion of the 95% confidence ellipse falls in the upper right quadrant or the lower left hand quadrant [35].

### Cost effectiveness acceptability curve

**Figure 3:**
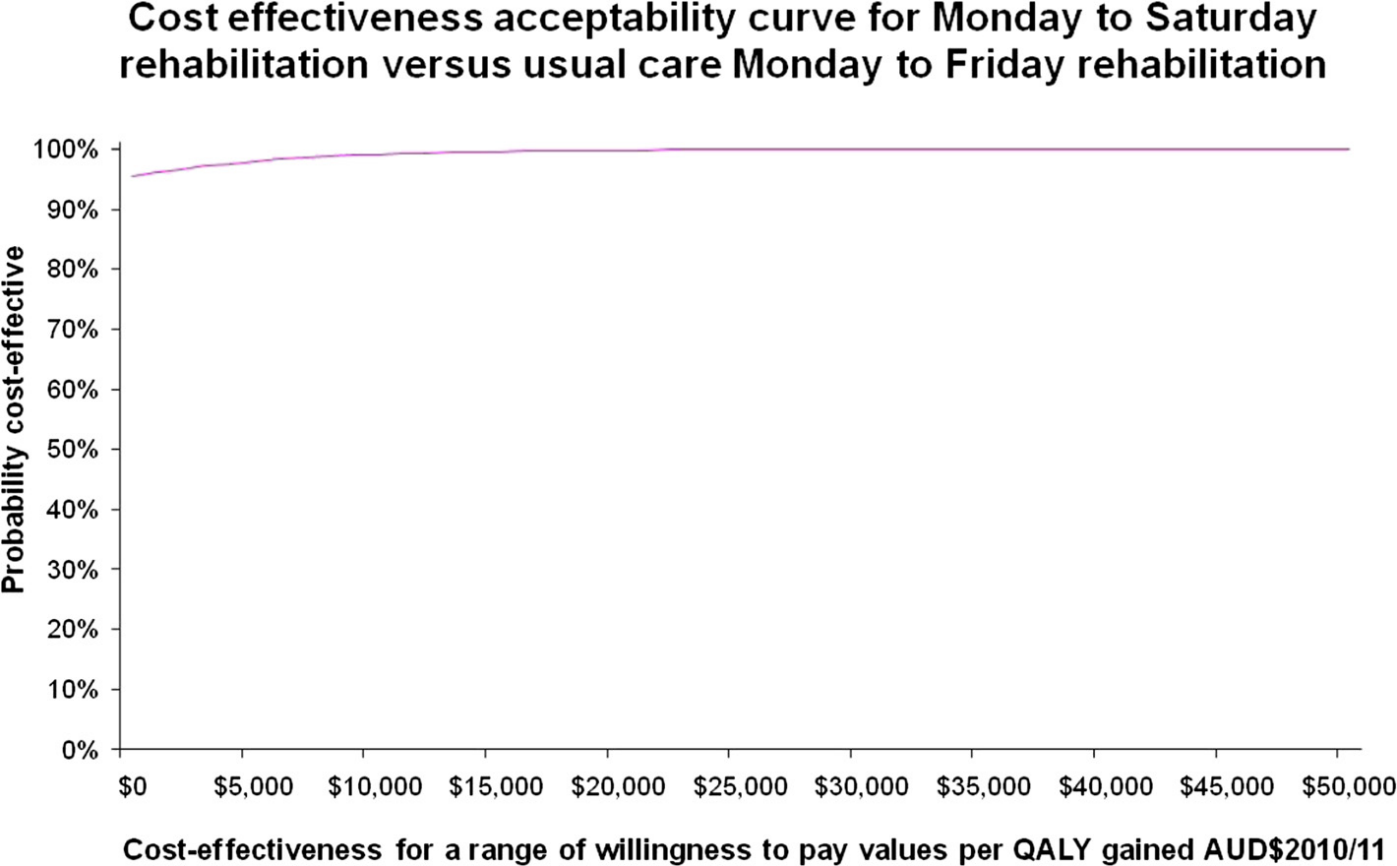
Cost effectiveness acceptability curve for Monday to Saturday rehabilitation versus usual care Monday to Friday rehabilitation for the probability of cost effectiveness (vertical axis) versus a range of cost effectiveness willingness to pay values (AUD$2010/2011) per quality adjusted life year (QALY) gained (horizontal axis).

**Figure 4:**
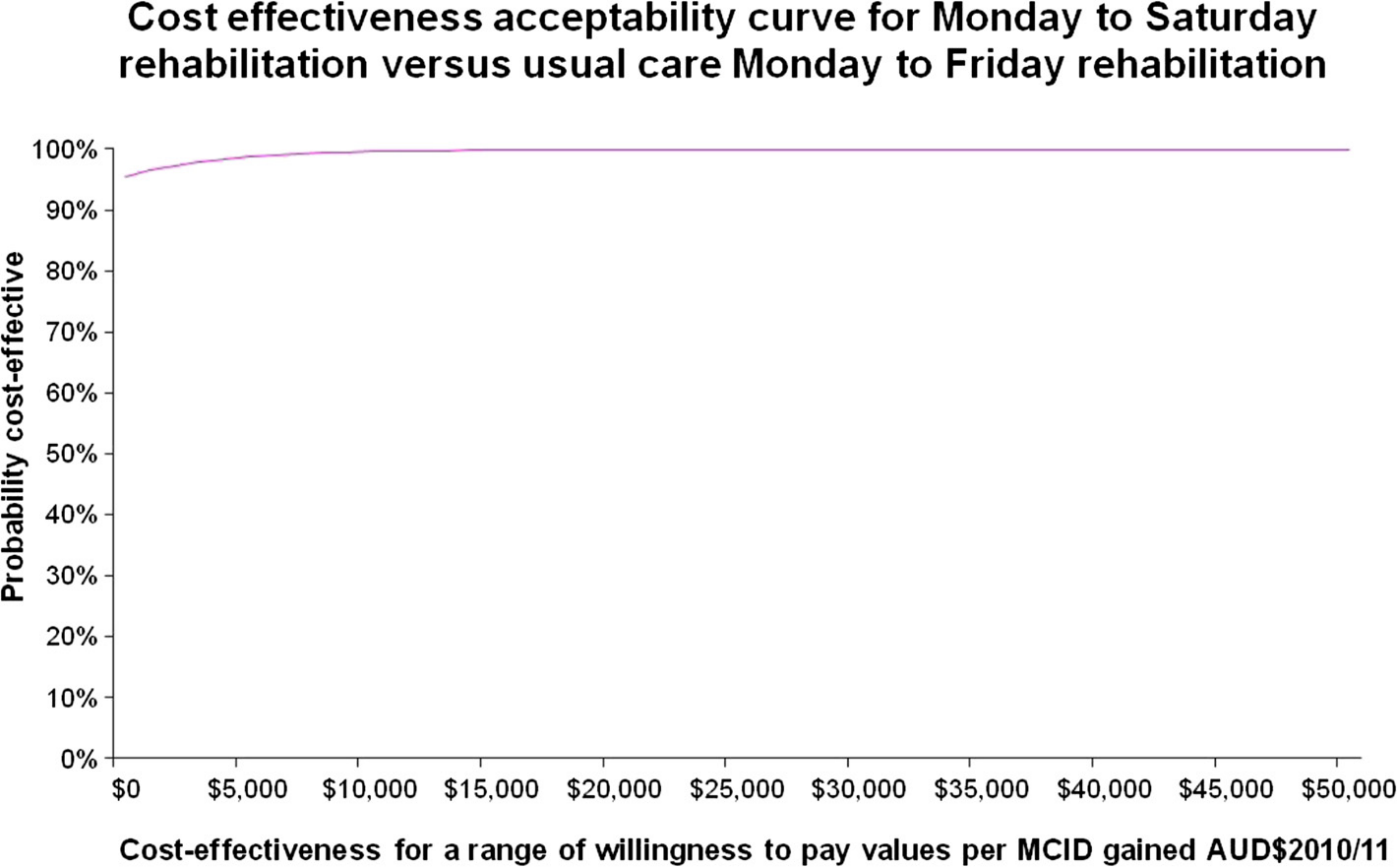
Cost effectiveness acceptability curve for Monday to Saturday rehabilitation versus usual care Monday to Friday rehabilitation for the probability of cost effectiveness (vertical axis) versus a range of cost effectiveness willingness to pay values (AUD$2010/2011) per minimal clinically important difference (MCID) in function gained (horizontal axis).

Figures 3 and 4 illustrate the cost effectiveness acceptability curves which show the willingness to pay values for a QALY gained (Figure 3) and for a MCID gained in functional independence (Figure 4). Both curves are relatively flat and show that with 99.3% certainty the willingness to pay for a QALY gained would be $13,000 and at 99.4% certainty the willingness to pay for a MCID gained in functional independence would be $10,000. Expressed another way, if the willingness to pay for a QALY gained or a MCID in functional independence was zero dollars then the probability of the intervention being cost effective is 96% and 95%, respectively.

### Sensitivity analysis

Removing the penalty loading for Saturday therapy staff, the health service cost during the initial rehabilitation admission and subsequent admissions in the 30 day period post discharge from rehabilitation was on average $15,824 (SD 13,970) for the intervention group and $17,532 (SD 17,108) for the control group, with a mean difference of -$1,708 (95% CI -3,651 to 235) in favor of the intervention group. This did not alter the results of the primary analysis.

## Discussion

Our results show a significant improvement in both outcome measures (EQ-5D-3L utility score and FIM functional score) as well as a difference in costs in favor of the provision of a rehabilitation service to inpatients on a Saturday in addition to Monday to Friday compared to Monday to Friday usual care rehabilitation alone. The confidence interval ellipses for the ICERs show that all the 50%, 75%, and most of the 95% confidence intervals fall within the bottom right hand quadrant suggesting that the intervention is likely to be dominant over the comparator of usual care. Therefore, from a health service perspective, the provision of a rehabilitation service to inpatients on a Saturday in addition to Monday to Friday compared to Monday to Friday rehabilitation alone, is likely to be cost saving per QALY gained and for a MCID gained in functional independence.

The National Institute for Health and Clinical Excellence (NICE) in the UK [36] reports a cost effectiveness threshold range of £20,000 (AUD$34,200) to £30,000 (AUD$51,400) per QALY gained [36] and there are similar values reported in Australia for the Australian Pharmaceutical Benefits Advisory Committee [37]. The results of this clinical trial report an average cost saving of over AUD$40,000 per QALY gained, in contrast to the above mentioned willingness to pay per QALY gained. If the willingness to pay in this study was AUD$50,000 per QALY gained or per MCID gained in functional independence, the probability of cost effectiveness in the intervention group approached 100%. Our results are consistent with a recent systematic review that reported a more intensive inpatient rehabilitation service can result in reduced cost to the health service, while improving patient outcomes [10].

This economic evaluation may have important implications for health services that offer inpatient rehabilitation, with potential to reduce costs per admission, improve patient outcomes and improve patient access. When considering the health service perspective, it is reported that in the inpatient rehabilitation setting the patient length of stay is the largest contributor to health care costs [38,39]. This may explain the likely reduction in cost for the intervention group, with an observed three day reduction in patient length of stay, over the rehabilitation admission and the 30 day readmission period. Policy makers may support this model of care with increased efficiency associated with cost savings, as it allows the same number of patient admissions to be managed at a lesser cost or may facilitate improved patient access to rehabilitation beds. This may lead to an improvement in the flow of patients through the health system and have a positive impact on the ‘bed block’ faced by acute wards [40,41]. For example, a 30 bed rehabilitation unit with an average length of stay of 29 days would have approximately 380 annual admissions; if the average length of stay reduced to 26 days, then annual admissions could potentially increase to 420.

Despite these benefits, implementation of this model of care also needs to consider workforce redesign. Traditionally, allied health clinicians work Monday to Friday, so the clinicians providing rehabilitation services may be reluctant to change their work practices by working on the weekend [8]. This may be negated by penalty rates providing an incentive to work on weekends. We note that we did not have a problem staffing the service in our clinical trial. Another issue for implementation involves who will pay and who will make the savings. This is about the redistribution of resources across budgets. Budget silos might mean that the costs will be incurred by allied health departments but the gains will be at the broader hospital level. If the funds come out of the smaller budgets of the departments providing the additional services (in this case physiotherapy or occupational therapy), it must be reconciled that these are not the budgets that accrue the overall savings generated at the hospital level.

The strengths of this economic evaluation are that it was completed alongside a blinded fully powered randomized controlled trial, it used an appropriate alternative intervention as 70% of Australian rehabilitation inpatient health services do not offer a weekend physiotherapy service [8], and it was reported according to the CHEERS checklist [see Additional file 1, [14]]. A limitation of this study includes the differing patient length of stay included in the calculation of health related quality of life and the functional status gained, because these measures were taken at admission and discharge from rehabilitation so that the mean change in clinical outcomes was for a different time period for each group. We have reported the mean change per day to address this limitation. In addition the calculation of incremental cost effectiveness ratios accounted for this variability since length of stay is the largest contributor to cost. While inclusion of patients with a cognitive impairment is considered a strength of this study, we are unable to report on the exact numbers of patients in this group. However, on admission to rehabilitation 5% (n = 54 of the 996 participants) did not complete the health related quality of life questionnaire and the main contributing factor was reduced cognition, as identified by the assessors. Other strengths of this study included access to complete clinical cost data on all patients across the two rehabilitation inpatient services, and inclusion of a range of rehabilitation diagnoses and patients with a language other than English as their first language. Therefore, we are confident that the results are generalizable across public acute phase inpatient rehabilitation settings. There were minor variations to the trial protocol. These included the use of multiple imputation rather than the carry forward technique for missing data, consistent with recent recommendations [27], as well as a reduced data collection period due to a higher than expected rate of participant recruitment. This study did not include the wider economic impact from a health system perspective during the rehabilitation inpatient admission, as well as the impact on the community once the patients are discharged from rehabilitation including return to work. As this economic evaluation did not use a health system perspective or report on long term economic outcomes post discharge, this warrants future research, which is planned.

## Conclusions

From a health service perspective, the provision of a rehabilitation service to inpatients on a Saturday in addition to Monday to Friday compared to Monday to Friday rehabilitation alone, is likely to be cost saving per quality adjusted life year gained and per minimal clinically important difference gained in functional independence.

## Abbreviations

ANCOVA: analysis of covariance; AUD$: Australian dollars; CEAC: cost effectiveness acceptability curve; CHEERS: Consolidated Health Economic Evaluation Reporting Standards; CI: confidence interval; EQ-5D-3L: EuroQol; ICER: incremental cost effectiveness ratio; ITT: intention to treat; MCID: minimal clinically important difference; NICE: National Institute for Health and Clinical Excellence; QALY: quality adjusted life year; RR: relative risk; SD: standard deviation; TTO: time trade-off.

## Competing interests

The authors declare that they have no competing interests.

## Authors’ contributions

NKB, JJW, NS and NFT conceived the clinical trial and economic evaluation design, analyzed the data, interpreted the results and drafted the manuscript. NFT and NKB are the guarantors. All authors had access to all of the data in the economic evaluation and can take responsibility for the integrity of the data and the accuracy of the data analysis. All authors read and approved the final manuscript.

## Pre-publication history

The pre-publication history for this paper can be accessed here:

http://www.biomedcentral.com/1741-7015/12/89/prepub

## Supplementary Material

Additional file 1 **Consolidated Health Economic Evaluation Reporting Standards (CHEERS) Checklist.** Items to include when reporting economic evaluations of health interventions. Click here for file
